# Oxidative Phosphorylation-Related Signature Participates in Cancer Development, and PTPRG Overexpression Suppresses the Cancer Progression in Clear Cell Renal Cell Carcinoma

**DOI:** 10.1155/2022/8300187

**Published:** 2022-11-10

**Authors:** Liang Huang, Yu Xie, Weiqing Han, Shusuan Jiang, Lingfeng Zeng

**Affiliations:** ^1^Department of Urology, The Affiliated Cancer Hospital of Xiangya School of Medicine, Central South University, Hunan Cancer Hospital, Changsha, Hunan 410013, China; ^2^Carol & Richard Yu Peritoneal Dialysis Research Centre, Department of Medicine & Therapeutics, Prince of Wales Hospital, Hong Kong; ^3^Li Ka Shing Institute of Health Sciences, Faculty of Medicine, The Chinese University of Hong Kong, Shatin, Hong Kong, China

## Abstract

Clear cell renal cell carcinoma (ccRCC) was a common cancer type diagnosed with frequent metastases, harboring an unfavorable therapeutic response, and results in a poor prognosis. More promising therapeutic targets are urgently required for treating ccRCC. This study was conducted to explore the role of oxidative phosphorylation in ccRCC development and reveal its clinical potential. We first identified oxidative phosphorylation-related clusters based on consensus clustering and validated their diversity in the genome instability, environmental infiltration, and immunosuppression by Gistic, ESTIMATE, GSVA, and TIDE web tools. We also compared their prognostic and clinical feature differences and predicted the IC50 level between the clusters using pRRophetic. Subsequently, we performed weighted gene coexpression network analysis to select cluster-related genes and performed functional analysis for them. The cluster-related genes were adopted to construct a risk score and nomogram for predicting patient prognosis with predictive accuracy evaluated. Finally, we performed lentivirus to induce ccRCC cell PTPRG overexpression and conducted western blot experiments to detect the critical protein expression of oxidative phosphorylation, apoptosis, cell cycle, and epithelial-mesenchymal transition processes. Also, the cell cycle and apoptosis level were evaluated by flow cytometry. As a result, we discovered that both the C1 cluster and high-risk group predicted patient survival with high accuracy and characterized lower survival rates, lower oxidative phosphorylation levels, higher immune infiltration, and malignant clinical features. Besides, we observed that overexpression of PTPRG activated oxidative phosphorylation and inhibited apoptosis. Its overexpression also depressed the epithelial-mesenchymal transition and promoted G1/S cell cycle arrest. Comprehensively, we confirmed the anticancer role of oxidative phosphorylation in ccRCC cells and discovered its association with immune and immunosuppression. PTPRG was also identified as a potential therapeutic target due to its multiple anticancer effects. We believe this study discovered a novel mechanism of ccRCC pathological progression and will provide promising targets for therapeutic strategy development.

## 1. Introduction

Renal cell carcinoma (RCC) is derived from the renal epithelium and is the most common cancer type in the kidney. The RCC consists of three major subtypes including clear cell RCC (ccRCC), papillary RC, chromophobe RC, and other rare subtypes with lower morbidity [1]. The ccRCC is the main subtype of renal cell carcinoma, taking over approximately 70 percent of all RCC subtypes. Though ccRCC can be treated at an early stage by routine strategies like surgical resection, the lethal metastases from the primary locus can be observed in a third of patients diagnosed with ccRCC [2], which usually causes an unfavorable prognosis without an effective approach to reverse.

The molecular characteristics of ccRCC have been widely investigated to affect the cancer process. For instance, the loss of chromosome 3p and VHL mutation has been found to frequently occur in ccRCC. These events caused the accumulation of HIF1/2*α* and trigged the hypoxia-related pathways [3]. Apart from the 3p chromosome loss and VHL mutation, the BAP1 mutation that occurred in 10% of the ccRCC has been proven a predictor of aggressive cancer cell phenotype. Based on these, the hypoxia-related pathway mutations have been discovered as a critical factor that interplays with many cancer hallmark changes, such as metabolic dysregulation and aberrated angiogenesis, to assist cancer progression, and the approaches targeting these mutations have achieved improved survival rates for the patients with ccRCC [4]. However, not all patients with ccRCC responded to the current therapeutic approaches. More discoveries of the ccRCC pathological mechanisms and promising strategies are still urgently required.

Oxidative phosphorylation is a common type of metabolism and is the main approach adopted by cells to produce sufficient ATP to support their growth. Compared with cancer-associated glycolysis, a hypoxia-related aberrate metabolism shifting cancer metabolism towards oxidative phosphorylation [5] has shown the ability to reverse the metastatic feature both in vivo and in vitro. However, many studies also demonstrated that oxidative phosphorylation is also an important risk feature with tumor-supportive roles in various cancers [6]. Therefore, oxidative phosphorylation seems to be a double-edged sword in cancers, and its extra role in the cancer process is still confusing. Currently, no clear evidence has presented the effects of oxidative phosphorylation on ccRCC cell progression, and an investigation of it is urgently required. Since hypoxia has been confirmed as a typical feature in ccRCC, the hypoxia-associated metabolic style like glycolysis also contributed [7] to cancer development. We preliminarily assumed a positive role of oxidative phosphorylation in ccRCC.

In this study, we identified oxidative phosphorylation-based subtypes and risk groups and investigated their prognostic value for ccRCC patients. Their cancerous biological diversity was explored via functional analyses, and the immunological heterogeneity as well as their potential as indicators of immunotherapeutic effects were evaluated. Importantly, we experimentally validated the influence of targeting the risk score key gene on ccRCC cell oxidative phosphorylation and its roles in affecting their malignant phenotypes. We believe this study promoted the understanding of oxidative phosphorylation in ccRCC and provided a novel target that will benefit the development of promising therapeutic strategies.

## 2. Materials and Methods

### 2.1. Data Acquisition

We retrieved the clear cell renal cell carcinoma (ccRCC) cohort from The Cancer Genome Atlas (TCGA) with their RNA sequencing data and clinical information. We also downloaded the oxidative phosphorylation, Gene Ontology (GO), and Kyoto Encyclopedia of Genes and Genomes (KEGG) data sets from Gene Set Enrichment Analysis (GSEA). The oxidative phosphorylation-related signature (OPS, HALLMARK_OXIDATIVE_PHOSPHORYLATION_v7.5.1.gmt) was selected as the input signature of our study analyses.

### 2.2. Division of Oxidative Phosphorylation-Related Clusters

We utilized the OPS as the input signature to perform a consensus clustering by the R package “ConsensusCluster” to divide the cohort into different clusters and used the PCA plot to test their data distance. We then plotted the Kaplan-Meier curve to evaluate the prognostic diversity of the clusters. We then used the “gistic2” R package [8] to estimate the copy number variation (CNV) of the clusters, as well as their intratumor heterogeneity, tumor mutation burden (TMB), cancer-testis antigen (CTA) score, and homologous recombination defect (HRD) differences [9]. Subsequently, the gene set variation analysis (GSVA) [10] was run to compare the hallmark gene set variation between the clusters.

### 2.3. Immunological Environment and Immunosuppressive Estimation

The “ESTIMATE” R package accessed the global immunological environment to calculate the immune score, stromal score, and tumor purity [11] differences of the clusters. The variation of 28 types of immunocyte infiltration levels was evaluated by the ssGSEA method. TIDE (Tumor Immune Dysfunction and Exclusion) [12] was used to determine levels of T cell dysfunction and immunosuppression in tumors, consisting of TID, IFNG, dysfunction, and exclusion score.

### 2.4. Clinical Exploration of the OPS Clusters

We evaluate the clinical value of the OPS clusters by comparing their differences in patient survival status, age, gender, tumor stage, and grade. For treatment consideration, we analyzed the IC50 of the drugs from the GDSC database using the R package “pRRophetic” [13] and selected several target drugs with a lower IC50 value in the C1 cluster. Further, we performed the weighted gene coexpression network analysis (WGCNA) based on the 25% top DEGs to identify the genes with a close correlation with the OPS clusters, and the inner gene correlation significance with the module was presented.

### 2.5. Functional Enrichment of the Cluster-Related Gene Sets

We applied the gene sets selected from the WGCNA module with the highest correlation with the phenotype to the functional analyses based on the GO (biological process, cellular component, and molecular function) and KEGG databases. These gene sets were obtained from the GSEA online database.

### 2.6. Construction of a LASSO-Based OPS Risk Score

We further filter the genes using the least absolute shrinkage and selection operator (LASSO) regression [14] to reduce the model complexity. The genes with the lowest deviance were selected to construct the risk score with their corresponding coefficients. The risk survival table presented the gene expression trend and survival status for patients arranged by their risk in both the training and validation cohort. The patients were divided into the low-risk and high-risk groups cut by the median risk score. We then plotted the Kaplan-Meier curves to access the prognostic value of the risk score and used ROC to evaluate the discrimination of the risk score for predicting patient survival. Also, we evaluate the risk score's capability of separating patient survival under clinical groups, including high/low age; female/male; G1, G2/G3, and G4 grades, and stage II/stage III and stage IV.

### 2.7. Clinical Significance of the Risk Score and the Nomogram Establishment

We plotted a Sanky plot to visualize the correlation among patient survival status, clusters, and risk groups. The risk score and the clinical parameters were applied to the univariate Cox analysis, and the parameters passing the univariate test were further input to the multivariate Cox analysis. We collected the two tests that were combined to establish a clinical nomogram to predict the 1-, 3-, and 5-year overall survival of ccRCC patients. A calibration curve was used to evaluate the calibration of the nomogram in predicting survival events. Besides, the clinical feature diversity between the risk groups was presented in the pie charts.

### 2.8. Cancerous and Immunological Heterogeneity between Risk Groups

The immunological heterogeneity was analyzed using ESTIMATE to calculate the environmental component inside the tumors to obtain the immune score, stromal score, and tumor purity for the risk groups. The enrichment degree of the cancer-related pathway and the 28 immunocyte infiltration was estimated using the ssGSEA calculation, and their correlation with the risk was presented. Then, the differences in the immune cell enrichment level differences were compared in the boxplot. Also, we performed ssGSEA to compare the glycolysis and the hypoxia to seek whether the hypoxia level was changed and the glycolysis was affected.

### 2.9. Cell Culture and Lentiviral Infection

We cultivated the 786-0 ccRCC cells in the cell incubator maintained at 37°C, 5% CO_2_ concentration, and the cells were fed with DMEM containing 10% FBS. We then collected the cells of the logarithmic growth phase to perform the experiments. The purchased PTPRG overexpression lentivirus was applied to infect the cells. After being seeded in the 12-well plates with medium, the cells were infected by the lentivirus and infection reagents for 16 hours, and the mixed medium was then replaced with fresh DMEM. After 48 hours, puromycin was added to screen the stable cells with fluorescent expression. The cells were then defined as the PTPRG-overexpression group (OE) and the control group (OE-NC).

### 2.10. Western Blot Analysis

We harvested the cell lyzed in the RIPA lysis. The lysed cells were treated with ultrasound sonication on ice and a metal bath, followed by a loading buffer mixture to prepare for electrophoresis. After electrophoresis and membrane transfer, the proteins on the membrane were blocked by skim milk for an hour. Then, we incubated the membrane overnight with the corresponding primary antibody (antibody brands listed in Supplementary Material S1). The next day, the membrane was washed with TBST three times and incubated by the secondary antibody and finally applied for chemiluminescence after TBST washing three times again.

### 2.11. CCK8 Assay

The harvested cells were resuspended with DMEM containing 10% FBS and seeded in the 96-well plates under 37°C, 5% CO_2_. After the cell adhered to the plate bottom, fluid was replaced with 100 *μ*l DMEM containing 10% CCK8 reagent inside each well, and all cells were cultivated in the incubator under the same conditions. The microplate reader examined each well's optical density at 0 h, 24 h, 48 h, and 72 h.

### 2.12. Flow Cytometry Detection of Cell Cycle and Apoptosis

For cell cycle detection, we digested and washed the cells with cold PBS and fixed them using 75% ethanol overnight. The fixed cells were centrifuged at 1,000 × g for 5 min at 4°C, and the supernatant was removed. The cells were resuspended using PI/RNase diluted in the staining buffer according to the protocol of the testing kit (Beyotime Biotechnology, Nantong, China). After incubation for 30 min from light, the cells were detected using the cytoFLEX Flow Cytometry System (Beckman-Coulter). As for apoptosis, the digested cells were washed with PBS and resuspended using binding buffer according to the apoptosis detection kit protocol (Beyotime Biotechnology, Nantong, China), after incubation with annexin-V and PI for 10 min from light, and the detection was performed using the Flow Cytometry System.

### 2.13. Statistical Analyses

The bioinformatic analyses were performed on the R platform. Consensus clustering divided the samples into clusters. Kaplan-Meier curves were plotted to evaluate the patient survival rate, and the results were tested by log-rank test. ROC was used to evaluate the survival prediction discrimination. Student *t*-tests compared the normally-distributed continuous parameters between two groups. Pearson's correlation coefficient quantified the correlation between genes and the WGCNA module. ANOVA compared the grouped results of the normally distributed parameters. *P* < 0.05 was considered statistically significant.

## 3. Results

### 3.1. OPS-Related Clusters Presented Different Prognosis and Genomic Status

**Figure 1:**
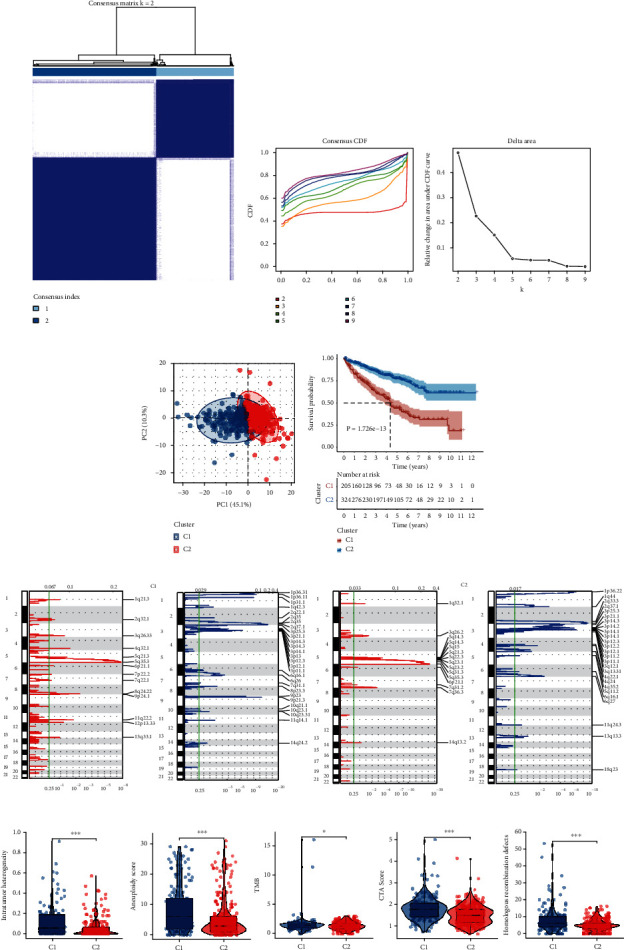
Consensus clustering and genomic instability analyses. The consensus clustering heat map (a), consensus CDF, and the relative change in area under the CDF curve (b) for the TCGA ccRCC cohort clustering. (c) The PCA plot depicts the sample distribution in the low dimension space divided by the clusters. (d) The Kaplan-Meier curve exhibited the time sequencing survival rate differences between the clusters. (e) The heat maps exhibiting the gene copy number amplification and deletion in each site of the chromosomes in each cluster. (f) Comparison of the genomic instability-related indexes between the clusters.

We obtained the OPS gene sets from GSEA and used them to conduct consensus clustering, which identified two clusters by the best *K* value according to the consensus CDF (Figures 1(a) and 1(b)). The PCA plot showed that the two clusters were separated when transformed into low dimensions (Figure 1(c)). We then evaluated the prognostic value of the clusters; the patients with the C1 cluster gained a lower survival rate (Figure 1(d)).

Subsequently, we explored the CNV differences between patients with different clusters. Obviously, the C1 clusters harbored higher gene amplifications than the C2 cluster (Figure 1(e)). When applying multiple indexes to evaluate their genome instability, the C1 cluster exhibited higher intratumor heterogeneity, aneuploid, TMB, CTA score, and HRD, demonstrating lower genomic instability in the C1 cluster. Moreover, we observed low levels of several pathways like oxidative phosphorylation and metabolism-associated pathways and various highly enriched cancerous or immunological pathways including IL6 JAK STAT3, TNFA, hypoxia, and glycolysis signaling pathways by the GSVA of cancer “hallmark” gene sets (Supplementary Material S2A), suggesting the association between the clusters and cancer immunology and glycolysis.

### 3.2. The Clusters Presented Higher Immunological Infiltration and Immunosuppressive Feature

**Figure 2:**
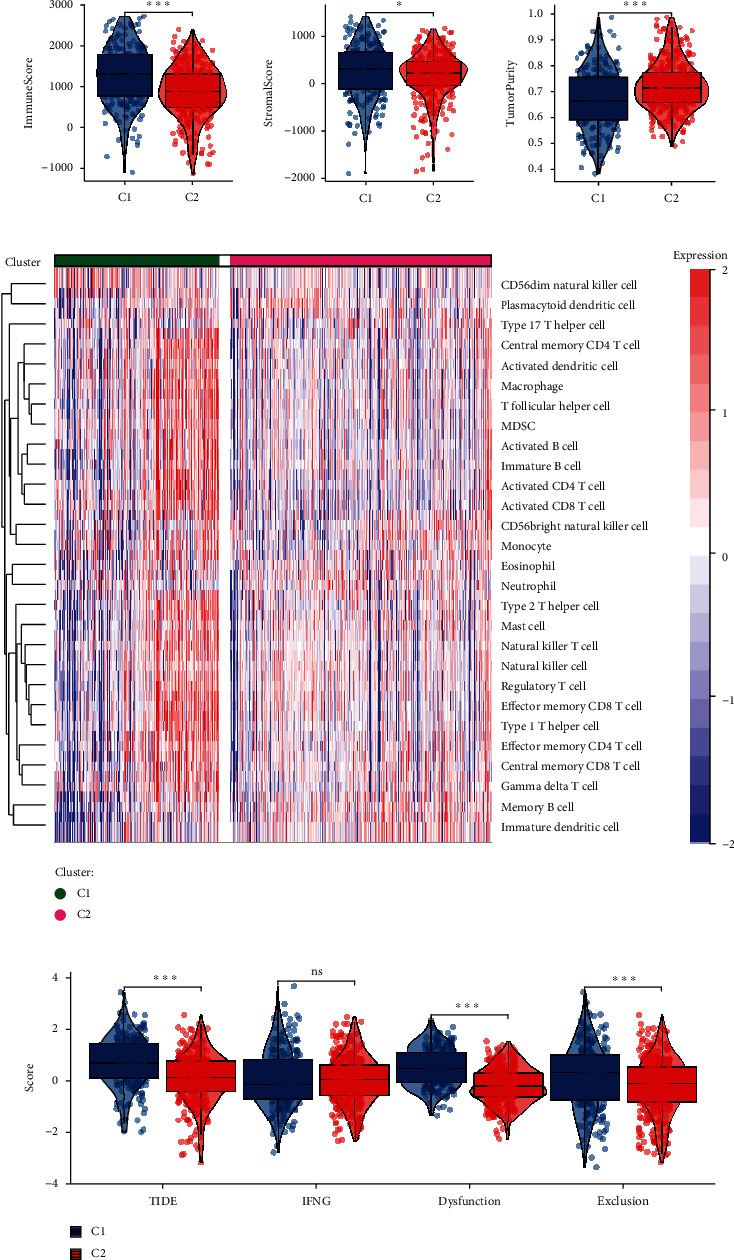
The immunological variation between subtypes. (a) Comparison of the immune score, stromal score, and tumor purity between the clusters. (b) The GSVA results in the immune cell infiltration levels of the clusters. (c) Comparison of TIDE analysis results between clusters including TIDE, IFNG, T cell dysfunction, and exclusion scores.

The ESTIMATE calculation was conducted to explicit the differences in the environment component between the clusters. We found that the C1 cluster presented a higher immune score and stromal score and lower tumor purity (Figure 2(a)). This was consistent with the GSVA results of the immune cell infiltration that most of the immunocytes were highly enriched in the C1 cluster (Figure 2(b)). Notably, MDSCs and regulatory T cells were also highly enriched in the C1 cluster, suggesting its potential immunosuppressive status. A further comparison of the TIDE-related indexes also demonstrated that the C1 cluster exhibited higher TIDE, T cell dysfunction, and immune exclusion levels (Figure 2(c)).

### 3.3. Patients with the C1 Cluster Presented a Higher Level of Unfavorable Clinical Features

**Figure 3:**
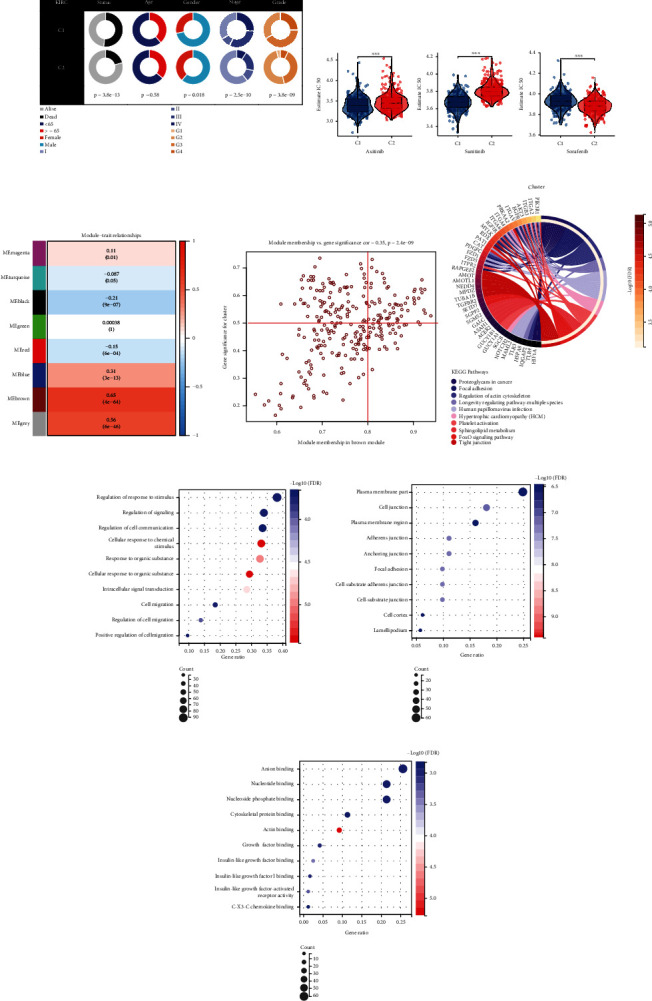
Clinical feature and WGCNA selection of the cluster-related genes. (a) The differences in clinical features between clusters C1 and C2. (b) Comparison of the GDSC compound IC50 of three drugs between clusters. (c) Correlation heat map of the WGCNA module-phenotype correlation (left) and the scatter plot showing the correlation between gene significance and module membership (right). Functional enrichment analyses of the KEGG (d), GO biological process (e), cellular component (f), and molecular function (g) gene sets.

To validate whether the clusters indicated clinical significance, we compared the clinical features of patients between the clusters. We observed that the C1 cluster was characterized by a significantly higher proportion of patients with dead status, male gender, and advanced stage and grade (Figure 3(a)). To develop potential drugs, we searched the GDSC to locate the drugs with a lower IC50 in the C1 cluster. As a result, three drugs, axitinib, sunitinib, and sorafenib, presented a lower IC50 significantly (Figure 3(b)).

We then performed WGCNA to filter the 25% top-variated genes firmly associated with the clusters. The genes were grouped into eight modules, and four modules presented a positive correlation with the cluster, and three modules exhibited a negative association; we selected the brown module with the highest correlation coefficients for downstream analysis, and this module presented a good correlation between the inner genes and the module phenotype (Figure 3(c)).

### 3.4. The Clusters-Related Genes Presented Different Biological Involvement

We performed functional enrichment analyses to investigate the biological activities that the genes with the highest correlation with clusters engaged. The KEGG pathway enrichment results exhibited the association between the genes and the tight junction. The enrichment results of the GO biological process, cellular component, and molecular function also presented the enrichment migration and adhesion junction-related gene sets (Figures 3(d)–3(g)), indicating that cluster-related genes were associated with cell migration and intercellular interactions.

### 3.5. Identification of an OPS Risk Score with Prognostic Value

**Figure 4:**
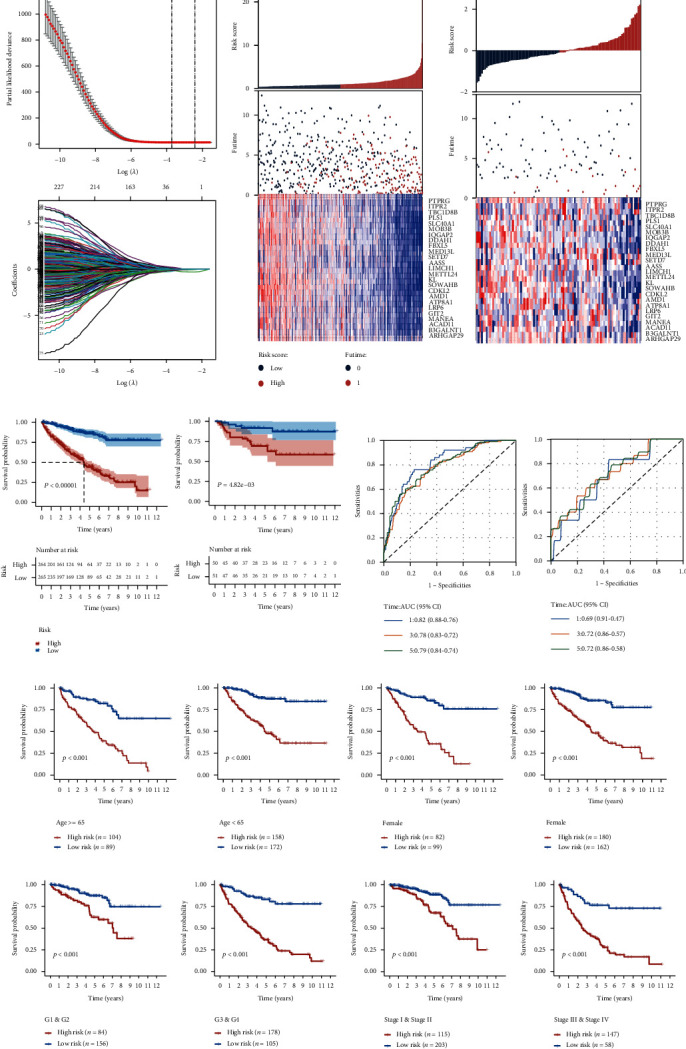
Construction of the risk score and its prognostic value. (a) The LASSO regression plot of the model partial likelihood deviance change (upper) and the regression coefficients (lower). (b) The heat map of the patient status time and key gene expression ranked by their risk score in the training (left) and the independent cohort (right). The Kaplan-Meier curves (c) and the ROC results (d) estimate the prognostic value of the risk score. (e) The Kaplan-Meier curve showing the survival analysis results under different clinical feature conditions presents the independent prognostic value of the risk score.

The cluster-associated genes were input to the LASSO regression [14] to construct a risk score. The 23 genes showing the lowest deviation were retained (Figure 4(a)). The 23 gene expression decreased as the risk arose in the training cohort, and most of them showed a similar trend in the independent cohort (Figure 4(b)). The risk score's capability to predict patient survival was tested, and the high-risk group predicted a lower survival rate in both the training and independent cohort (Figure 4(c)), and their performance estimation results confirmed its predictive accuracy (Figure 4(d)). Also, we noticed that the risk score can predict patient survival independent of age, gender, tumor grade, and stage (Figure 4(f)), indicating the important prognostic value of the risk score.

### 3.6. Potential for Clinical Application of the Risk Score

**Figure 5:**
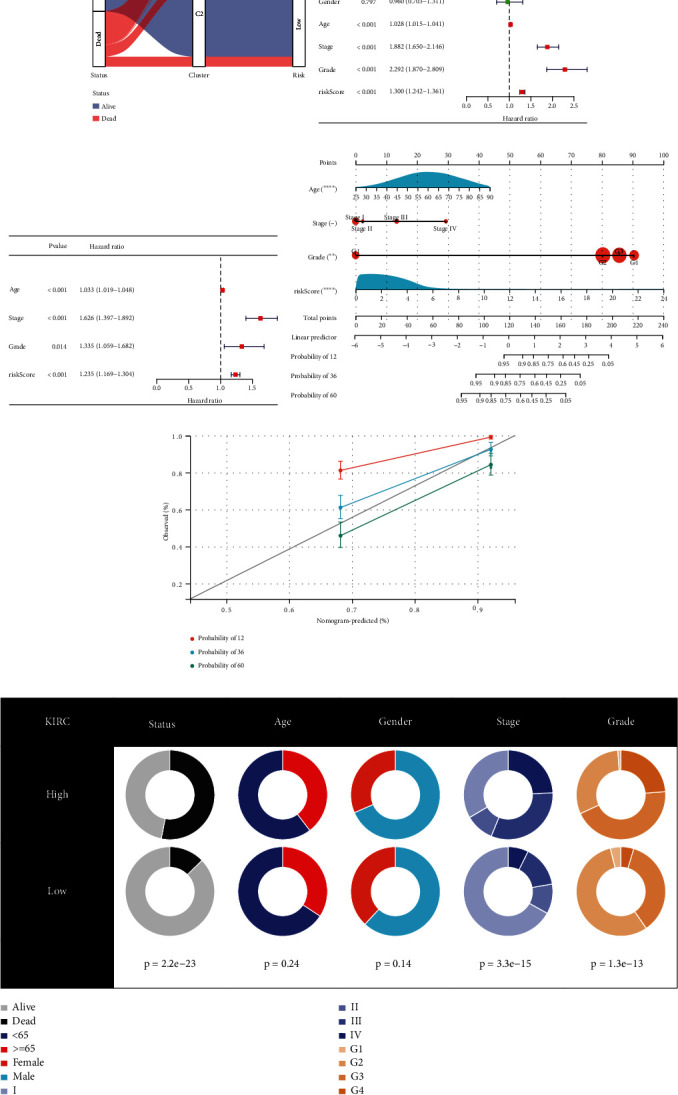
Establishment of the clinical nomogram. (a) The “Sanky” plot of the association among patient survival status, clusters, and risk groups. The forest plots of the clinical features were filtered by the univariate (b) and multivariate (c) Cox regression. (d) Constructed clinical nomogram consists of age, stage, grade, and risk score for predicting patient overall survival. (e) Calibration curve for validating the calibration of the nomogram's predictive ability for patient survival. (f) The differences in the clinical features between the low-risk and high-risk groups.

The “Sanky” plot showed the phenotype correlation among patient status, cluster, and risk group; most of the high-risk group patients harbored C1 cluster feature and dead status (Figure 5(a)). The univariate and multivariate Cox analyses screened out several independent predictors, including age, stage, grade, and risk score (Figures 5(b) and 5(c)). They were gathered to construct a clinical nomogram to predict the overall survival of one year, three years, and five years with a good calibration (Figures 5(d) and 5(e)). Also, the high-risk group patients were characterized by more dead cases, high stage, and grade (Figure 5(f)), indicating their unfavorable events.

### 3.7. The High-Risk Group Showed Diverse Immune Cell Infiltration and Cancer Hallmark-Related Pathway Enrichment

**Figure 6:**
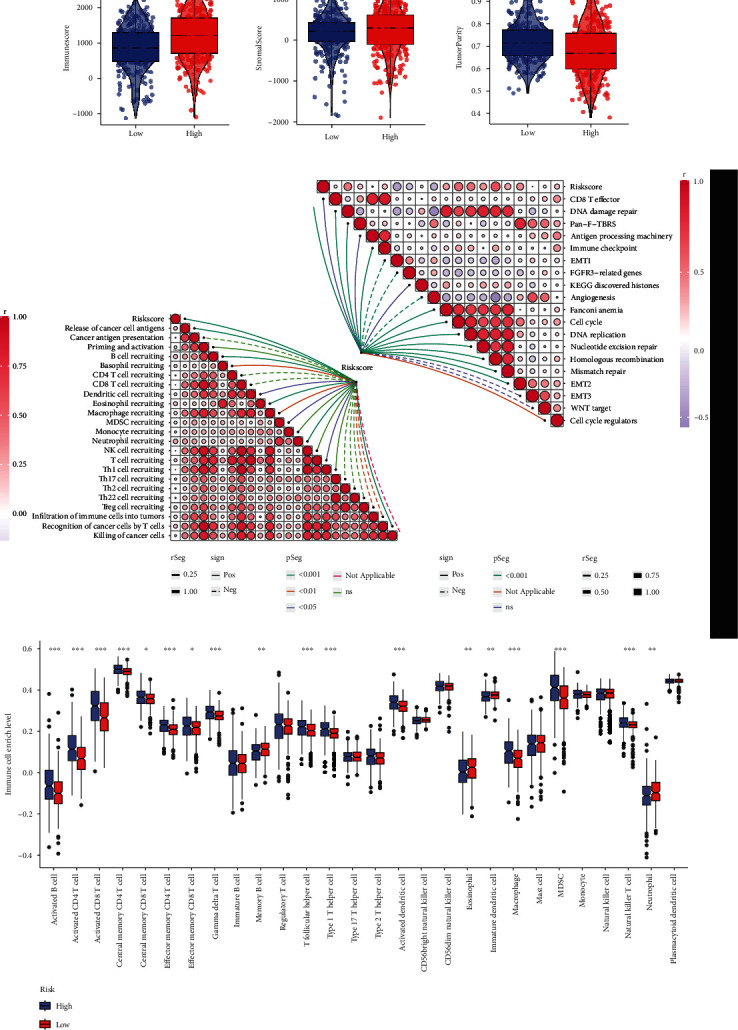
Immune infiltration and cancer hallmark heterogeneity between risk scores. (a) Evaluation of the environmental component differences between risk groups by immune score, stromal score, and tumor purity. (b) The correlation between patient risk, cancer hallmark gene set expression (right), and anticancer immunity cycle gene sets (left). (c) The immune cell enriches differences between risk groups presented in a box plot.

ESTIMATE was used to evaluate the environmental component differences between the risk groups. Similar to the C1 cluster, the high-risk group also exhibited higher immune score, stromal score, and lower tumor purity (Figure 6(a)). Also, we used GSVA to calculate the enrichment of cancer hallmarks and cancer antitumor immunity cycle gene sets [15]. The risk score was positively associated with the cell cycle and a series of nuclei-repair-related gene sets. For the antitumor immunity cycle, the risk score was positively correlated with recruiting MDSC and macrophage while negatively associated with recruiting CD4 and CD8 positive cells (Figure 6(b)). The box plot also exhibited the highly enriched MDSC and macrophage in the high-risk group (Figure 6(c)). These suggested the environmental complexity and possible immunosuppressive status in the high-risk group. Besides, we compared the level of glycolysis and hypoxia between the clusters and risk groups by ssGSEA, while no differences were observed (Supplementary Materials S2B and S2C).

### 3.8. Overexpression of PTPRG Increased the Oxidative Phosphorylation and Inhibited Cell Apoptosis

**Figure 7:**
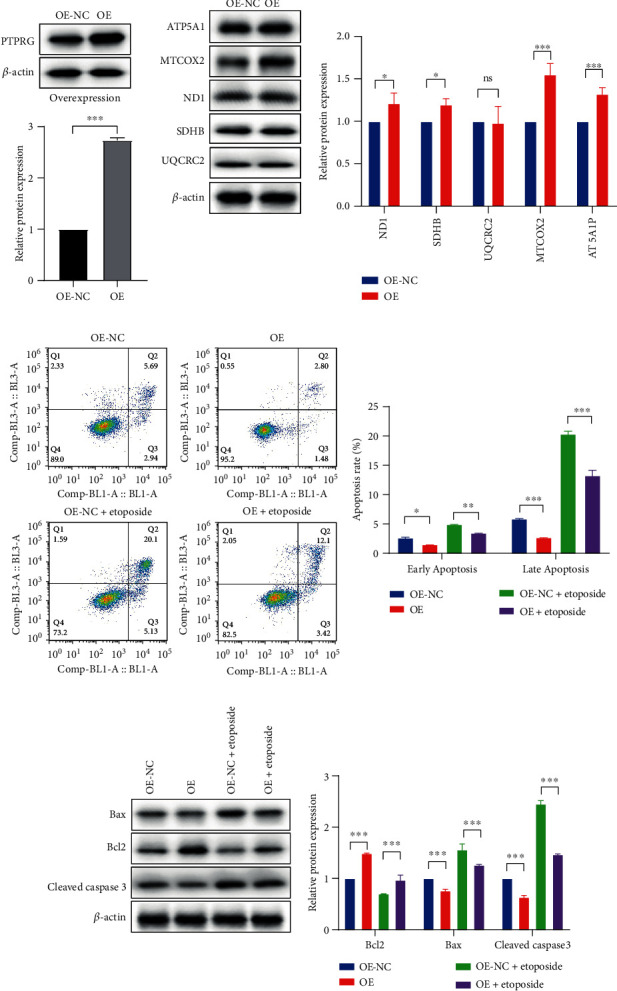
PTPRG overexpression activated oxidative phosphorylation and inhibited apoptosis. (a) The western blot bands of the PTPRG protein expression in OE-NC and OE groups (upper) and the statistical comparison bar plot of the results (lower). (b) The western blot bands showing the expression of the oxidative phosphorylation-related proteins (left) and their statistical comparison (right). (c) The scatter plots of the apoptosis analyzed by flow cytometry and their statistical comparison (right). (d) The western blot bands show the apoptosis-related protein expression differences between OE-NC and OE groups treated with or without etoposide (left) and their statistical comparison (right).

We used lentivirus transfection to investigate the role of PTPRG overexpression in ccRCC cells. The western blot result showed a significantly increased PTPRG protein expression of the OE group (Figure 7(a)). To explicit whether PTPRG overexpression activated the oxidative phosphorylation of ccRCC cells, we detected the protein levels of ATP5A1, MTCOX2, ND1, SDHB, and UQCRC2, and we observed the increased expression of ND1, SDHB, ATP5A1, and especially MTCOX2 protein significantly (Figure 7(b)).

We then analyzed the apoptosis level of the ccRCC cells in the OE and OE-NC groups as well as cells treated with the proapoptosis drug etoposide using flow cytometry. The results demonstrated that the overexpression of PTPRG decreased the early and late apoptosis rates in both untreated and etoposide-treated ccRCC cells significantly (Figure 7(c)). We further observed elevated antiapoptosis protein Bcl2 in the OE group and decreased expression of the antiapoptosis proteins Bax and cleaved caspase 3 for both cells treated with and without etoposide (Figure 7(d)). These discoveries indicated that PTPRG overexpression in ccRCC cells activated the oxidative phosphorylation activity and decreased their apoptosis.

### 3.9. PTPRG Overexpression Arrests the Cell Cycle Transition from G1 to S Phase and Inhibited the EMT Process

**Figure 8:**
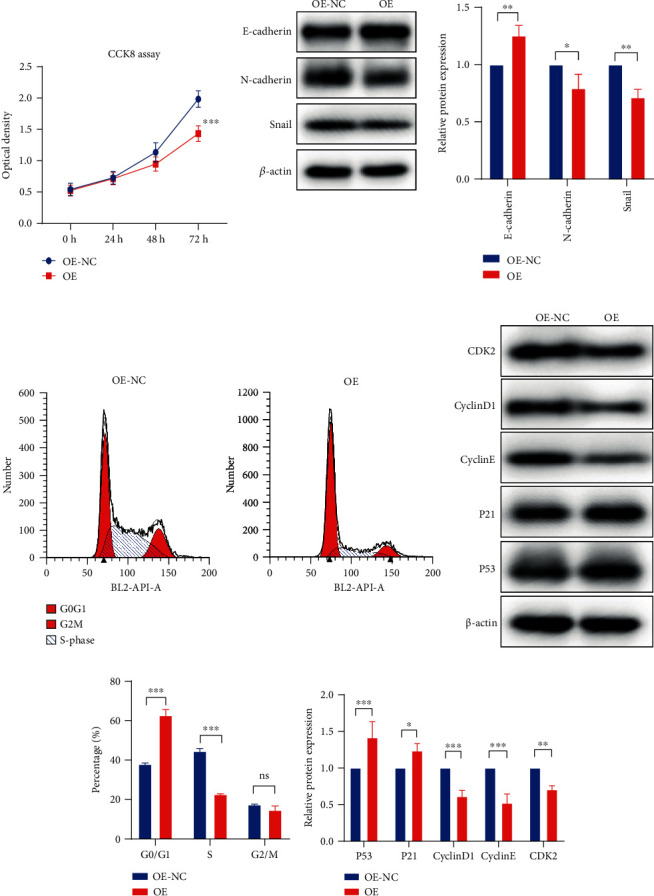
PTPRG overexpression depressed EMT and mediated cell cycle arrest. (a) The optical density detected in 0 h, 24 h, 48 h, and 72 h indicates the cell viability in CCK8 assay. (b) The western blot bands of the EMT-related protein expression (left) and their statistical comparison (right). (c) The flow cytometry results of the G0/G1, S, and G2/M phase cell cycle proportion. (d) The western blot bands of the G1/S phase transition-related protein expression. (e, f) The statistical comparisons of the cell cycle proportion (e) and G1/S phase transition-related protein expression (f) between OE and OE-NC groups.

We first performed the CCK8 cell viability test for the function of PTPRG overexpression in ccRCC cells. As Figure 8(a) shows, the optical density of OE-NC exceeded that of the OE group at 72 h, indicating the depressed cell viability in the OE group. Also, we observed inhibited EMT activity according to the increased E-cadherin and decreased N-cadherin and Snail protein expression (Figure 8(b)) in the PTPRG OE group. Then, flow cytometry was used to detect the cell cycle of the ccRCC cells.

As the results exhibited, the OE group cells presented higher G1/G0 phase and lower S and G2/M phases (Figure 8(c)). The level of critical proteins in regulating the cell cycle was detected, and we noticed that CDK2, CyclinD1, and CyclinE were downregulated in the OE group (Figure 8(d)). The results demonstrated that PTPRG overexpression could prevent ccRCC progression by inhibiting EMT and G1/S cell cycle transition.

## 4. Discussion

Oxidative phosphorylation has double-faced roles in cancer progression, and its effects on ccRCC remain unclear. Here, we identified clusters and risk groups associated with oxidative phosphorylation levels. We noticed that the cluster or high-risk group with a low level of oxidative phosphorylation predicted worse survival, indicating the anticancer property of oxidative phosphorylation in ccRCC. Also, the ability to predict patient survival demonstrated the robustness of our clusters and risk model for patient prognosis indication. To our best knowledge, this is the first study revealing the association between oxidative phosphorylation and ccRCC prognosis based on large cohort analysis.

This study also discovered the association between oxidative phosphorylation and immunocyte infiltration. Notably, we noticed the highly infiltrated MDSC in the high-risk group and cluster 1. In the mouse breast cancer model, high oxidative phosphorylation levels in the MDSC were found to drive the immunosuppressive function of the myeloid-derived suppressor cell (MDSC) [16]. In human ovarian cancer cells, the MDSC with immunosuppressive characteristics presented energetic metabolism, and reduced oxidative phosphorylation was observed with decreased expression of immunosuppressive markers [17]. In contrast, an MDSC dynamic metabolic flux analysis discovered that the immunosuppressive MDSC exhibited cancer-related Warburg effects, with a low oxygen consumption rate and depressed oxidative phosphorylation level [18]. Similarly, in the microenvironment of the three cancer type animal model, the MDSC without immunosuppressive function presented elevated rates of oxidative phosphorylation compared to the control group [19]. The variated results in different cancers suggested the complex and context-dependent role of oxidative phosphorylation in regulating the immunosuppressive role of MDSC. In ccRCC, no study concerning the role of oxidative phosphorylation in MDSC has been reported so far. Interestingly, the hypoxia and glycolysis levels did not variate between the clusters and risk groups, suggesting the nonglycolysis mediated MDSC infiltration in cluster 1 and the high-risk group, and this may be directly attributed to the oxidative phosphorylation that the high level of oxidative phosphorylation depressed the infiltration of MDSC. However, the limitation is that the underlying mechanism of the MDSC infiltration inhibition by oxidative phosphorylation remains unknown. Anyway, our study first reveals the highly infiltrated MDSC in ccRCC, while the detailed mechanism of oxidative phosphorylation in MDSC and how it trigged the MDSC-mediated immunosuppression deserved further investigation.

Protein tyrosine phosphatase receptor type G (PTPRG) is a member of the tyrosine phosphatase family regulating various biological processes in cancer [20]. Its anticancer function has been discovered in many cancers. In nasopharyngeal carcinoma, PTPRG inhibited the Akt signaling pathway mediating growth and invasion of cancer cells [20]. In breast cancer, PTPRG was found to inhibit tumor formation [21], and miR-19b was validated to support cell migration by suppressing PTPRG expression [22]. Additionally, PTPRG was also found to suppress the progression of tumors not only in solid tumors but also in chronic myeloid leukemia [23]. The various evidence supported that PTPRG was a tumor suppressor gene in many cancers. However, the anticancer effects of PTPRG on ccRCC are still undiscovered. Here, we experimentally confirmed the anticancer role in ccRCC cells for the first time for its overexpression depressed the EMT processes and induced cell cycle arrest. Also, the upregulation of the oxidative phosphorylation pathway proteins in cells with overexpressed PTPRG elucidated the association between PTPRG and oxidative phosphorylation, though their extract interaction still awaits discovery. Interestingly, except for a tumor suppressor within the cancer cells, PTPRG was also reported as a key node of the immunophenotype regulation hub, indicating its potential to improve immunotherapeutic effects [24].

Comprehensively, we identified oxidative phosphorylation-associated clusters and risk groups with high performance to predict patient survival accurately. The cluster and high-risk group with a low oxidative phosphorylation rate were characterized by poor prognosis and malignant clinical features, complex tumor environment, and higher immunosuppressive levels. We also confirmed that overexpression of PTPRG increased protein expression of the oxidative phosphorylation pathway, as well as the decreased EMT and arrested cell cycle. This indicated the protective role of oxidative phosphorylation and PTPRG high expression in ccRCC. We think this study clarifies the interaction between oxidative phosphorylation and inner tumor processes and provides a promising therapeutic target for ccRCC patients.

## Data Availability

TCGA expression data and clinical information can be downloaded from https://portal.gdc.cancer.gov/. The oxidative phosphorylation, GO, and KEGG gene sets can be obtained from http://www.gsea-msigdb.org/gsea/index.jsp. The TIDE analysis was analyzed on http://tide.dfci.harvard.edu/. The GDSC compound data can be found on https://www.cancerrxgene.org/. The codes can be retrieved from the corresponding author.

## Abbreviations

ccRCC: Clear cell renal cell carcinoma
RCC: Renal cell carcinoma
TCGA: The Cancer Genome Atlas
GO: Gene Ontology
KEGG: Kyoto Encyclopedia of Genes and Genomes
OPS: Oxidative phosphorylation-related signature
CNV: Copy number variation
TMB: Tumor mutation burden
CTA: Cancer-testis antigens
HRD: Homologous recombination defects
GSVA: Gene set variation analysis
TIDE: Tumor Immune Dysfunction and Exclusion
WGCNA: Weighted gene coexpression network analysis
LASSO: Least absolute shrinkage and selection operator
OE: Overexpression group
OE-NC: Control group
MDSC: Myeloid-derived suppressor cell
PTPRG: Protein tyrosine phosphatase receptor type G.

## Additional Points

*Contribution to the Field Statement*. Clear cell renal cell carcinoma is a common subtype of renal cell carcinoma. Though clear cell renal cell carcinoma patients can benefit from early diagnosis and treatment, many patients were diagnosed with metastases and suffered poor prognoses. The cancer marker-based treatment has improved the therapeutic efficacy for many cancers, but the promising strategy for treating late-stage clear cell renal cell carcinoma is still lacking. In our study, we confirmed the anticancer role of oxidative phosphorylation in clear cell renal cell carcinoma, which has not been reported previously. We discovered that cancers with low oxidative phosphorylation are characterized by poor prognosis, malignant clinical information, genomic instability, and complex environment infiltration, demonstrating the importance of oxidative phosphorylation in cancer development. Also, we found that PTPRC overexpression activated oxidative phosphorylation, inhibited cancer cell epithelial-mesenchymal transition, and mediated cell cycle arrest, resulting in cancer progression suppression. Therefore, our study discovered the novel biological mechanism involved in the cancer process and provided promising targets for developing an effective therapeutic strategy for patients suffering from limited treatment response.

## Supplementary Materials

Supplementary 1 Supplementary Material S1: the information of the antibodies used in this study.

Supplementary 2 Supplementary Material S2: the GSVA results of the cancer hallmark gene set and comparison of hypoxia and glycolysis between clusters and risk groups.
