# Supplementary material for: Oxidative Phosphorylation-Related Signature Participates in Cancer Development, and PTPRG Overexpression Suppresses the Cancer Progression in Clear Cell Renal Cell Carcinoma
Source: J Immunol Res. 2022 Nov 10;2022:8300187. doi: 10.1155/2022/8300187 (PMC9673183; doi:10.1155/2022/8300187)
Supplement: Supplementary 1 — Supplementary Material S1: the information of the antibodies used in this study. [file 8300187.f1.docx]

Supplementary Material S1 **The information of the antibodies used in this study**

PTPRG, Invitrogen, cat.# PA5-15524;

β-actin, Proteintech, 66009-1-Ig;

ND1, Proteintech, 19703-1-Ig;

SDHB, Proteintech, 10620-1-Ig;

UQCRC2, Proteintech, 14742-1-Ig;

MTCOX2, Proteintech, 55070-1-Ig;

ATP5A1, Proteintech, 14676-1-Ig;

Bcl2, Cell Signalling Technology, cat.#15071;

Bax, Cell Signalling Technology, cat.#89477;

Cleaved Caspase 3(cell signalling technology, cat.#9661)

p53, Cell Signalling Technology, cat.#2527;

p21, Cell Signalling Technology, cat.#2947;

ClyclinD1, Cell Signalling Technology, cat.#55506;

CyclinE, Cell Signalling Technology, cat.#20808;

CDK2, Cell Signalling Technology, cat.#18048;

E-cadherin, Cell Signalling Technology, cat.#14472;

N-cadherin, Cell Signalling Technology, cat.#13116;

Snail, Cell Signalling Technology, cat.#3879.
