# Supplementary figures and images for: Oxidative Phosphorylation-Related Signature Participates in Cancer Development, and PTPRG Overexpression Suppresses the Cancer Progression in Clear Cell Renal Cell Carcinoma
Source: J Immunol Res. 2022 Nov 10;2022:8300187. doi: 10.1155/2022/8300187 (PMC9673183; doi:10.1155/2022/8300187)

**A**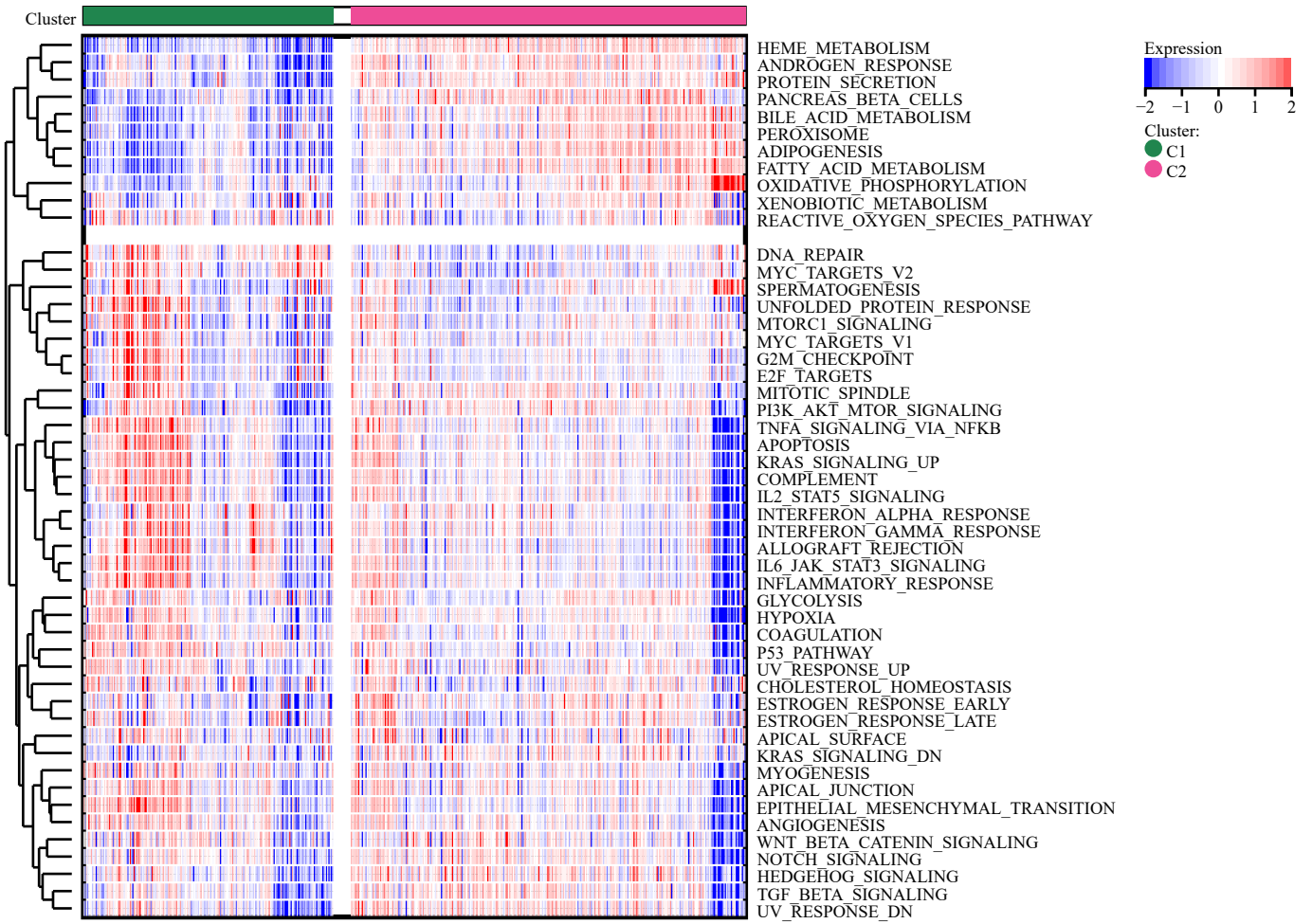**B**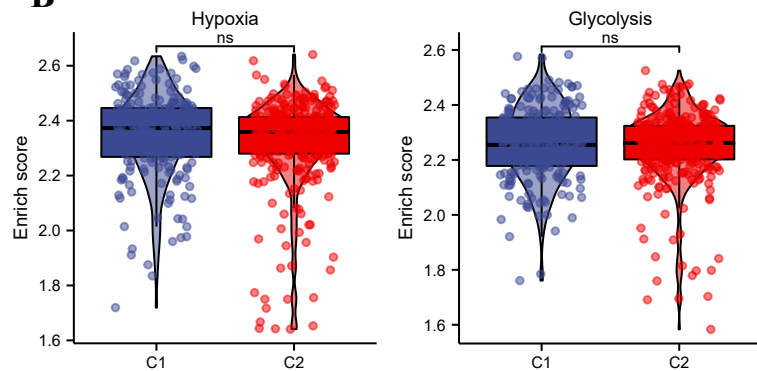**C**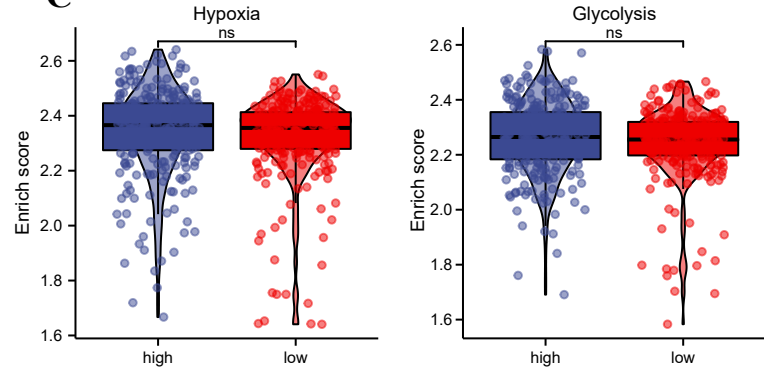

Supplement: Supplementary 2 — Supplementary Material S2: the GSVA results of the cancer hallmark gene set and comparison of hypoxia and glycolysis between clusters and risk groups. [file 8300187.f2.pdf]
